# Sex and SP-A2 Dependent NAD(H) Redox Alterations in Mouse Alveolar Macrophages in Response to Ozone Exposure: Potential Implications for COVID-19

**DOI:** 10.3390/antiox9100915

**Published:** 2020-09-25

**Authors:** He N. Xu, Zhenwu Lin, Chintan K. Gandhi, Shaili Amatya, Yunhua Wang, Lin Z. Li, Joanna Floros

**Affiliations:** 1 Britton Chance Laboratory of Redox Imaging, Department of Radiology, Perelman School of Medicine, University of Pennsylvania, Philadelphia, PA 19104, USA; hexu2@pennmedicine.upenn.edu (H.N.X.); zxl13@psu.edu (Z.L.); 2 Department of Pediatrics, Center for Host Defense, Inflammation, and Lung Disease, College of Medicine, The Pennsylvania State University, Hershey, PA 17033, USA; cgandhi@pennstatehealth.psu.edu (C.K.G.); samatya@pennstatehealth.psu.edu (S.A.); ywang@psu.edu (Y.W.); 3 Departments of Pediatric and Obstetrics and Gynecology, College of Medicine, The Pennsylvania State University, Hershey, PA 17033, USA

**Keywords:** optical redox imaging, nicotinamide adenine dinucleotide (NAD(H)), oxidized flavoprotein containing flavin adenine dinucleotide (FAD), redox ratio, surfactant protein A2 (SP-A2), surfactant protein A, macrophage activation, ozone, redox heterogeneity, innate immunity, lung

## Abstract

Co-enzyme nicotinamide adenine dinucleotide (NAD(H)) redox plays a key role in macrophage function. Surfactant protein (SP-) A modulates the functions of alveolar macrophages (AM) and ozone (O_3_) exposure in the presence or absence of SP-A and reduces mouse survival in a sex-dependent manner. It is unclear whether and how NAD(H) redox status plays a role in the innate immune response in a sex-dependent manner. We investigated the NAD(H) redox status of AM from SP-A2 and SP-A knockout (KO) mice in response to O_3_ or filtered air (control) exposure using optical redox imaging technique. We found: (i) In SP-A2 mice, the redox alteration of AM in response to O_3_ showed sex-dependence with AM from males being significantly more oxidized and having a higher level of mitochondrial reactive oxygen species than females; (ii) AM from KO mice were more oxidized after O_3_ exposure and showed no sex differences; (iii) AM from female KO mice were more oxidized than female SP-A2 mice; and (iv) Two distinct subpopulations characterized by size and redox status were observed in a mouse AM sample. In conclusions, the NAD(H) redox balance in AM responds to O_3_ in a sex-dependent manner and the innate immune molecule, SP-A2, contributes to this observed sex-specific redox response.

## 1. Introduction

In the lung, more than 80% of cells in the bronchoalveolar lavage (BAL) are macrophages [1,2]. Macrophages engulf and digest cellular debris, cancer cells, foreign substances, and bacteria by phagocytosis. Macrophages play a critical role in nonspecific defense (innate immunity) and are important as antigen presenters to T cells [3]. Macrophages also play important pro-inflammatory and anti-inflammatory roles depending on the specific conditions and modulate immune reactions through cytokine release [4].

Metabolism has profound influence on macrophage polarization/activation and pathogenesis [5]. Macrophage activation is critically supported by metabolic shifts. Pro-inflammatory macrophages have an anaerobic metabolic profile based on glycolysis whereas anti-inflammatory macrophages generally rely on oxidative phosphorylation (OXPHOS) for energy generation [6,7]. Nicotinamide adenine dinucleotide (NAD^+^ and the reduced form NADH, denoted as NAD(H)) is an essential co-enzyme for hundreds of reactions within the cell [8]. A change in the NAD^+^/NADH ratio (redox shift) can profoundly affect metabolism, including reactions of glycolysis and the tricarboxylic acid cycle (TCA) and OXPHOS. NAD(H) is also a key mediator in metabolic network, and an NAD^+^-coupled redox status shift has been demonstrated upon activation of macrophages [9]. Ozone (O_3_) exposure accelerates glycolysis in macrophages which requires constant re-oxidation of NADH [10].

As an essential electron donor, NADH (and the reduced nicotinamide adenine dinucleotide phosphate (NADPH)) is intrinsically fluorescent and emits blue light (~450 nm) upon the excitation by UV light (~360 nm). Another intrinsically fluorescent molecule, flavin adenine dinucleotide (FAD), is an oxidized cofactor of flavoproteins that catalyze a wide range of biological redox reactions. FAD emits green light (~520 nm) when being excited by blue light (~430 nm). NADH and FADH_2_ (the reduced form of FAD) are fed into the electron transport chain in mitochondria to generate adenosine-5′-triphosphate by OXPHOS. Pioneered by Chance et al. [11,12,13,14,15], optical redox imaging (ORI) detects the intrinsic fluorescence signals of NADH and Fp (oxidized flavoproteins containing FAD) and provides the optical redox ratio, Fp/NADH or the normalized redox ratio, Fp/(NADH+Fp). The redox ratio provides a measure of mitochondrial redox state. It has been shown that Fp/NADH and Fp/(NADH+Fp) linearly correlate with the biochemically determined NAD^+^/NADH [16] and the mass-spectrometry determined NAD^+^/(NADH+NAD^+^) [17,18], respectively. ORI is sensitive to cellular metabolic changes and reactive oxygen species (ROS) generation [19,20] and has been widely applied to study cellular metabolism and bioenergetics in both normal and diseased tissues [21,22,23]. ORI is also sensitive to or correlates with the genetic status and responds to genetic modulations (overexpression and knockdown) of oncogenes [17,24,25,26,27,28,29]. ORI of the fluorescence intensity and lifetime of NADH and FAD has also been shown to identify macrophage activation phenotypes [9,30] and detect metabolic heterogeneity of macrophages within the tumor microenvironment [30]. 

Surfactant protein A (SP-A), an innate immune molecule in the lung, has a significant impact on the alveolar macrophages (AM) proteome [31,32] and bacterial phagocytosis [33]. Ozone (O_3_)-induced oxidative stress in mice with or without SP-A exhibited a sex-dependent effect on mouse survival after bacterial infection [33,34] in lung pneumonia dissemination [35,36], and on markers of inflammation and oxidative stress in the BAL [37]. However, humans (unlike rodents) have two functional genes, *SFTPA1* and *SFTPA2*, encoding SP-A1 and SP-A2 proteins, respectively; and a number of genetic variants have been identified for each gene [38]. SP-A1 and SP-A2 have been shown to differentially affect the AM proteome [39,40], mouse survival and lung function [41,42]. Moreover, after O_3_-induced oxidative stress, the AM miRNome (full spectrum of micro RNAs expressed in the genome) in mice carrying either SP-A1 or SP-A2 was differentially affected in a sex-dependent manner, with SP-A2 having a major impact on males [43]. In the present study, we built on these previous findings in an attempt to better understand the mechanisms of O_3_-induced oxidative stress in the presence or absence of SP-A2, from the perspective of AM metabolism using optical redox imaging. Specifically, we found that ozone exposure altered the NAD(H) redox status of mouse AM in a sex-dependent manner in the presence of SP-A2, and SP-A2 may play a role in females in counteracting O_3_-induced oxidation by modulating the NAD(H) redox status.

## 2. Materials and Methods

### 2.1. Study Mice

All the mice used in the present study were 9–12 weeks of age. The SP-A2 mice used were generated on the C57BL6/J SP-A (KO) background [44]. The 1A^0^ variant, the most common SP-A2 genetic variant in humans, is used for the current study. The animals were raised and maintained under approved housing in a pathogen-free condition at the Penn State College of Medicine animal facility. Both males and synchronized females (with regard to the estrous cycle) were used in this study. The Penn State University College of Medicine Institutional Animal Care and Use Committee approved all procedures involving animals (ethical protocol code 44968). 

In this study, a total of 31 mice (16 KO mice and 15 SP-A2 mice) were used for ORI. Among the 16 SP-A KO mice, 7 mice (4 males and 3 females) were exposed to filtered air (FA) as control, and 9 mice (5 males and 4 females) were exposed to ozone. Among the 15 SP-A2 mice, 6 mice (3 males and 3 females) were exposed to filtered air, and 9 mice (5 males and 4 females) were exposed to ozone (Table S1). 

### 2.2. Ozone Exposure and AM Isolation

SP-A2 (1A^0^) or KO male and female mice were exposed to 2 ppm ozone at room temperature and 50% humidity for 3 h, as described previously [45]. All ozone exposures and filtered air exposures were conducted in parallel [46]. Mice were sacrificed at 4 h after exposure and subjected to BAL for isolation of alveolar macrophages, as described previously [39]. In brief, AM were obtained by performing BAL with 1 mM EDTA in PBS, using a volume equal to lung capacity (0.5 mL × 6 times, a total of 3 mL). The fluid, each 0.5 mL, was instilled and withdrawn 3 times with chest massage during withdrawal. The lavage was kept on ice while being transferred from Penn State University to the University of Pennsylvania for optical redox imaging as described below. The time lapse from BAL to arriving at the optical redox imaging lab was ~3.5 h.

### 2.3. Optical Redox Imaging and Data Analysis

AM in lavage were spun and resuspended in RPMI1640 supplemented with 10% FBS. The cell resuspension was then seeded on a 35 mm glass-bottom dish (Mat-Tek or CellVis) and incubated at 37 °C, 5% CO_2_ for ~3 h. Approximately 10 min before imaging, the cells were rinsed with PBS (with Ca^2+^ and Mg^2+^) twice, followed by the addition of 1 mL of Live Cell Imaging Solution (LCIS, Life Technologies) spiked with glucose (final concentration 11 mM) for imaging.

For FA-exposed SP-A2 mice, after imaging of the redox baseline, redox plasticity [47] was imaged by sequentially adding mitochondrial metabolic perturbation drugs to first uncouple mitochondrial oxidative phosphorylation then inhibit the mitochondrial complex I and III. Trifluoromethoxy carbonylcyanide phenylhydrazone (FCCP, 0.5 µM) was used as the uncoupler and a mixture of rotenone (1 µM) and antimycin A (1.25 µg/mL) (ROTAA) was used as the inhibitor. Images were taken approximately 3–5 min post addition of each chemical agent. 

Mitochondrial ROS was measured by adding MitoSOX red (2 µM final) to dishes and incubating at 37 °C (protected from light) for 10 min. Dishes were then rinsed once with PBS and added 1 mL LCIS supplemented with 11 mM glucose for imaging. 

A Zeiss wide field microscope (Axio Observer 7) set at 37 °C was used for imaging. Images were taken using a 20X objective (0.8 NA). NADH signals were collected through the following optical bandpass filters: excitation (Ex) 370–400 nm and emission (Em) 414–450 nm. Fp signals were acquired with Ex 450–488 nm and Em 500–530 nm bandpass filters. Transmitting (white) light was used to locate and focus on regions of interest to avoid photo-bleaching. Five to ten randomly selected non-overlapping fields of view (FOVs) per dish were imaged with shading corrections on the fly. The image size is 1920 × 1216 (pixel size 0.29 µm^2^). For imaging MitoSOX signals, the cells were excited at 385 ± 15 nm and detected at 595 ± 15 nm.

Acquired images were processed with a customized routine of Matlab^®^. Details of data analysis can be found in our previous reports [25,47]. In brief, the cell-free background signals were subtracted from each of the raw images. A signal-to-noise ratio threshold of 7.5 was further applied to exclude the low-intensity pixels, where noise is defined as the standard deviation in the background. The redox ratio Fp/(NADH+Fp) images were generated pixel-by-pixel using NADH and Fp images. The net mean values of each of the redox indices (NADH, Fp, and the redox ratio) of each FOV were averaged to obtain the dish mean and further averaged across dishes to obtain group means (Table S1). The redox indices for AM from one KO male (mouse ID 12273) in the ozone-exposed group deviated by two standard deviations from the group means and thus were considered an outlier and excluded for comparisons between groups.

Statistical analysis was performed with either Student’s *t*-test assuming unequal variance or ordinary one-way ANOVA with post-hoc Bonferroni correction for multiple comparisons using PRISM 8. For each redox index, we pooled the data from all groups (sex, exposure, and SP-A2 status) for ANOVA to determine the sex, ozone, and SP-A2 effects on the NAD(H) redox status. The results are presented as mean ± standard deviation (SD). *p* < 0.05 is considered statistically significant. Significant differences are displayed as: * *p* < 0.05, ** *p* < 0.01, *** *p* < 0.001, and **** *p* < 0.0001. 

## 3. Results

### 3.1. Metabolic Responses of AM

**Figure 1:**
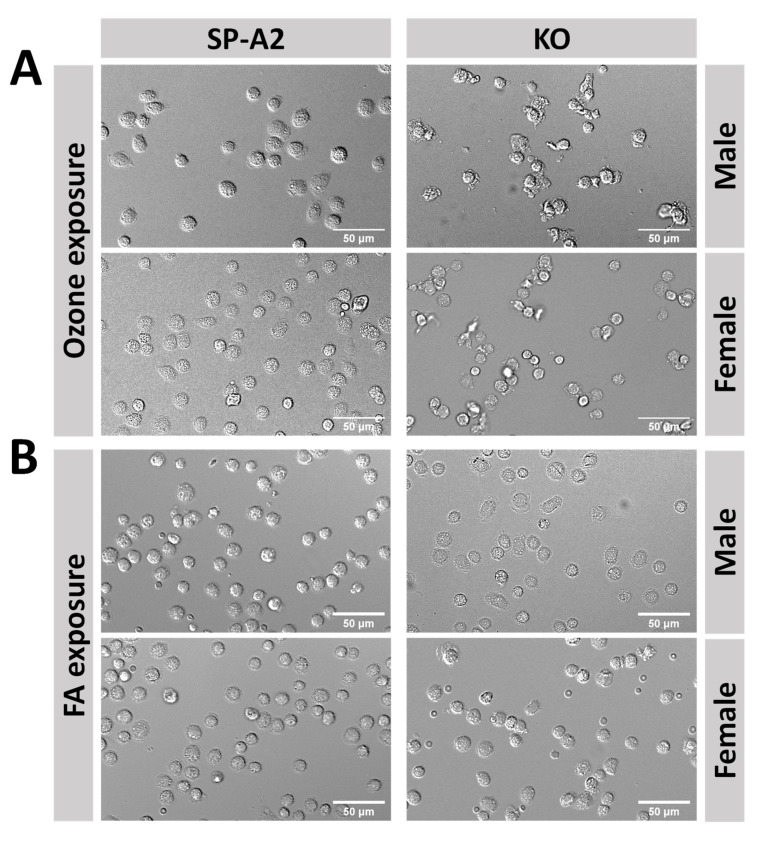
Typical morphological appearance of alveolar macrophages (AM) from SP-A2 and knockout (KO) mice exposed to ozone (**A**) or FA (**B**) showing distressed cell morphology of AM from both male and female KO mice only after ozone exposure.

In KO mice remarkable changes were readily observed in cell morphology of AM after ozone exposure as shown in the representative white light images (Figure 1A). For both males and females, AM from SP-A2 mice appeared to be rounder, fuller, and well separated, whereas AM from KO mice appeared to be irregular shaped and tended to aggregate. For AM from SP-A2 and KO mice under the FA condition, such differences were almost unnoticeable (Figure 1B).

**Figure 2:**
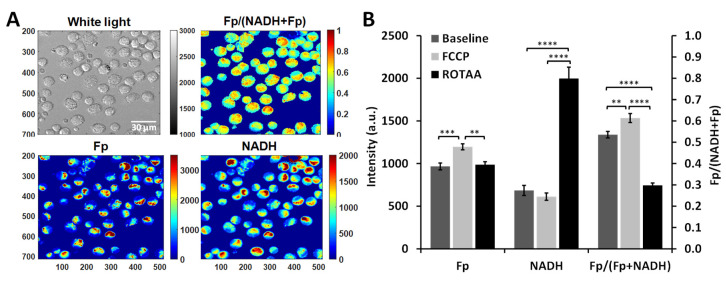
Optical redox imaging (ORI) of alveolar macrophages (AM) is sensitive to metabolic/redox perturbations. (**A**) Typical white light and redox images of AM from a female SP-A2 mouse (control group, filtered air (FA)-exposed). The grey/color bars to the right side of white light and the Fp and NADH images represent signal intensity in arbitrary units; whereas the color bar for the redox ratio Fp/(NADH+Fp) image represents the redox ratio ranging from 0 to 1. (**B**) Quantitative redox responses of AM from SP-A2 females to the metabolic perturbations by FCCP and ROTAA (Mean ± SD), confirming that ORI is sensitive to the redox changes under the experimental settings. ** *p* < 0.01, *** *p* < 0.001, and **** *p* < 0.0001.

We first confirmed that ORI was sensitive to metabolic and redox changes under the experimental settings. After imaging the redox baseline (representative images shown in Figure 2A), we sequentially perturbed the redox status of AM from SP-A2 mice (FA-exposed) with a mitochondrial oxidative phosphorylation uncoupler (FCCP) followed by mitochondrial inhibitors (ROTAA). Treating cells with a mitochondrial oxidative phosphorylation uncoupler such as FCCP should result in a more oxidized state (i.e., higher redox ratio), whereas treating cells with mitochondrial inhibitors such as rotenone (complex I inhibitor) and/or antimycin A (complex III inhibitor) should result in a more reduced state (i.e., lower redox ratio). Using AM from SP-A2 females as an example, after sequentially adding FCCP and ROTAA and imaging accordingly, the ORI signals yielded the expected changes as shown in Figure 2B. Specifically, upon FCCP treatment, the Fp signal significantly increased and the macrophages became significantly more oxidized with a higher optical redox ratio (the oxidized extreme in this study) compared to the baseline; upon subsequent ROTAA treatment, the Fp signal lowered to its baseline level, the NADH significantly increased and the macrophages became significantly more reduced with a lower redox ratio (the reduced extreme) compared to both baseline and FCCP treatment. AM from the male SP-A2 mice exhibited similar changes.

### 3.2. Impact of Sex, Ozone, and SP-A2 on AM Redox Status

We investigated the effect of three main factors, sex, O_3_ exposure, and SP-A2 (presence or absence), on the NAD(H) redox status of AM. As ozone-induced oxidative stress was shown to differentially affect the AM miRNome of male and female SP-A2 mice, with males being impacted significantly [43], the focus of the present study was on the redox status of AM from the SP-A2 mice. 

#### 3.2.1. Sex Differences under the FA Condition

**Figure 3:**
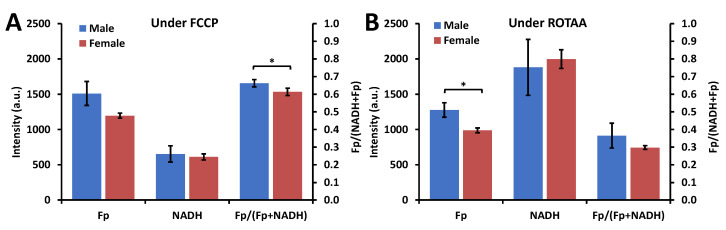
Sex-associated redox difference observed when AM from SP-A2 mice were subject to metabolic stress under the FA condition. (**A**) AM in the oxidized extreme when treated with mitochondrial oxidative phosphorylation uncoupler FCCP; (**B**) AM in the reduced extreme when treated with mitochondrial complex I inhibitor (rotenone, ROT) + mitochondrial complex III inhibitor (Antimycin A) (Mean ± SD). * *p* < 0.05.

**Figure 4:**
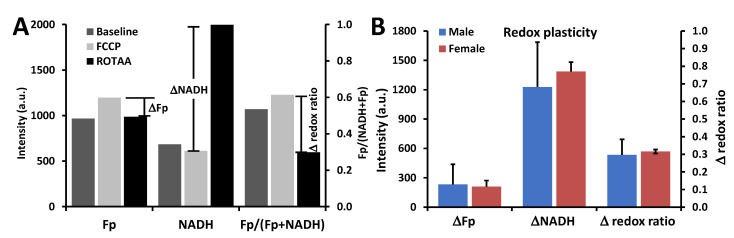
No significant sex-dependent difference in the redox plasticity of SP-A2 AM under the FA condition. (**A**) Illustration of calculations of redox plasticity, which is the difference between the oxidized (FCCP) and reduced (ROTAA) extremes of the redox indices. The calculation was first performed for AM from each mouse then averaged across the AM samples for each sex group; (**B**) Redox plasticity of AM from SP-A2 males and females (Mean ± SD).

Under the FA condition, no significant sex differences were observed in any of the baseline redox indices (Fp, NADH, and the redox ratio) for AM from SP-A2 mice (discussed below). However, when AM from SP-A2 mice were under metabolic stress, the sex difference in the redox indices became significant (Figure 3). Specifically, upon FCCP treatment, which uncouples mitochondrial OXPHOS and pushes the cells to the oxidized extreme, AM from the males were relatively more oxidized than that of the females (*p* < 0.05) (Figure 3A). Upon ROTAA treatment, which inhibits mitochondrial complex I and III and pushes the cells to the reduced extreme, AM from the females had relatively less Fp value (*p* < 0.05) (Figure 3B). Besides the redox baseline measures, redox plasticity provides additional information. By calculating the difference in AM from SP-A2 mice between the oxidized and reduced extremes of each redox index as shown in Figure 4A, ΔFp (representing the Fp plasticity), ΔNADH (representing the NADH plasticity), and Δ redox ratio [47,48], we quantified the redox plasticity for each redox index and found no significant redox plasticity difference between AM from males and females (Figure 4B). These measurements show that sex difference under the FA condition becomes significant only when AM were subject to metabolic stress and under unperturbed conditions no significant inherent differences could be detected between AM from SP-A2 males and females.

#### 3.2.2. Ozone Effect

**Figure 5:**
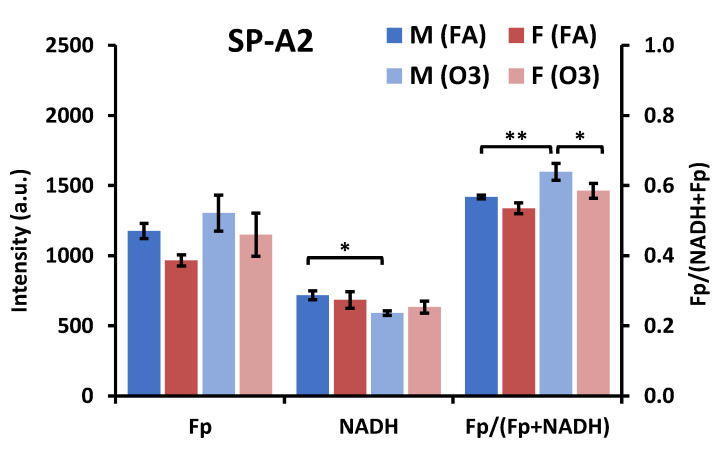
Sex-associated redox difference of AM in SP-A2 mice exposed to FA or O_3_ (Mean ± SD). F-female; M-male. * *p* < 0.05, ** *p* < 0.01.

As noted above and shown in Figure 5 there was no significant difference in any of the redox indices under the FA condition. In response to ozone exposure the NAD(H) redox status of AM from female SP-A2 mice was not significantly affected (Figure 5). However, in males the NADH level of AM was significantly lowered, indicating that NADH was consumed to counteract ozone-induced oxidative stress. As a result, the cells were shifted to a more oxidized state indicated by significantly higher redox ratio in comparison with that under the FA condition (Figure 5). The redox ratio of AM from male mice was significantly higher compared to AM from female mice under the O_3_ condition. Thus, after O_3_ exposure, sex-associated redox differences became significant between males and females, as ozone exposure shifted AM from male SP-A2 mice to a more oxidized state with insignificant impact on the AM redox status of female SP-A2 mice. 

**Figure 6:**
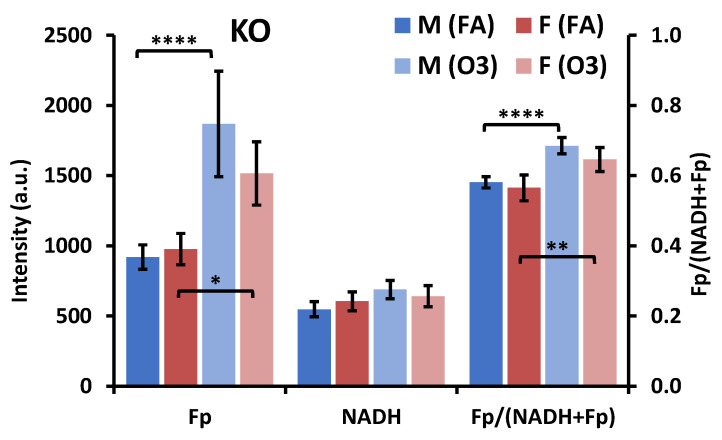
Sex-associated redox difference of AM in KO mice exposed to FA or O_3_ (Mean ± SD). F-female; M-male. * *p* < 0.05, ** *p* < 0.01, and **** *p* < 0.0001.

In KO mice, no significant redox difference was observed in AM between male and female mice under the FA condition (Figure 6), similar to that of AM from SP-A2 mice. However, in response to O_3_ exposure, the Fp significantly increased and so did the redox ratio, regardless of sex in KO mice (Figure 6).

**Figure 7:**
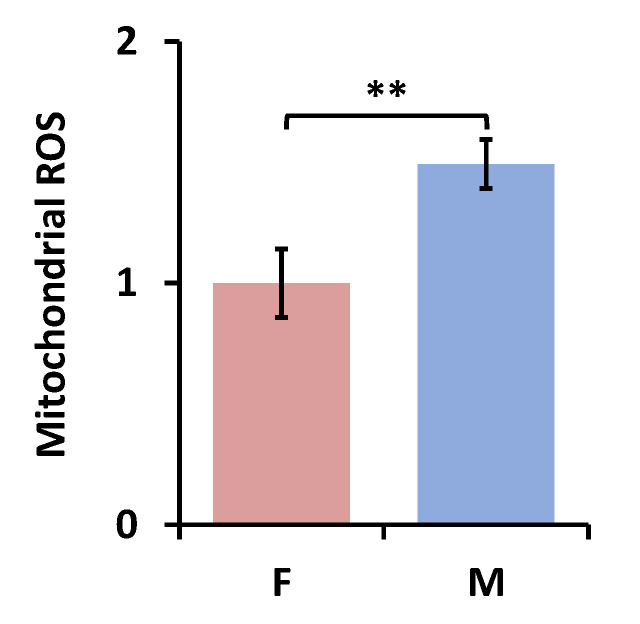
Sex-associated difference in the mitochondrial ROS level (normalized to females) in AM from SP-A2 mice exposed to ozone (Mean ± SD). F-female; M-male. ** *p* < 0.01.

As reported in our previous study [19], the NAD(H) redox status is intricately related to reactive oxygen species (ROS) generation. Since AM from male SP-A2 mice were in a more oxidized state compared to females after ozone exposure, we expected to see a difference in their ROS levels. As shown in Figure 7, under the ozone condition, we indeed found that AM from male mice had ~30% higher mitochondrial ROS compared to females (*p* < 0.01), positively correlating with the higher redox ratio in AM from males. Since we did not find any significant differences in the redox indices between males and females in KO mice upon ozone exposure (Figure 6), we did not measure ROS in these groups.

#### 3.2.3. SP-A2 Impact on NAD(H) Redox Status

**Figure 8:**
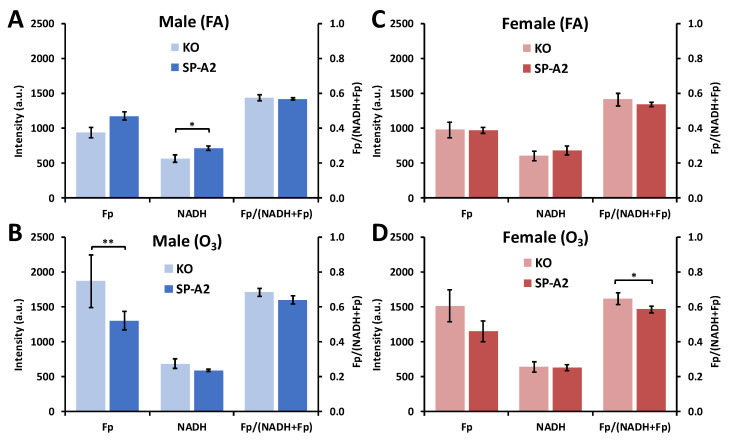
SP-A2-associated redox differences in AM from male (**A**,**B**) and female (**C**,**D**) mice exposed to FA (**A**,**C**) or O_3_ (**B**,**D**) (Mean ± SD). * *p* < 0.05, ** *p* < 0.01.

We next investigated the impact of SP-A2 on NAD(H) redox status of AM. For male mice, under the FA condition, AM from SP-A2 mice compared to those of KO had ~26% higher NADH (*p* = 0.016), ~26% higher Fp (*p* > 0.99) and similar redox ratio (Figure 8A), indicating that SP-A2 may affect mitochondrial density in AM since the majority of ORI signals come from mitochondria. In contrast, after ozone exposure, AM from SP-A2 males had approximately 30% lower Fp (*p* = 0.0021), ~14% lower NADH (*p* = 0.15), and ~6% lower redox ratio (*p* = 0.12) (Figure 8B), indicating that SP-A2 may affect AM’s anti-oxidative stress capacity. For female mice, under the FA condition, there is no significant difference in any of the redox indices between AM from SP-A2 and KO mice (Figure 8C); however, under the ozone condition, compared to KO, AM from SP-A2 were in a more reduced state, indicated by a 10% lower redox ratio (*p* = 0.020), despite the fact that no significant change was observed in either Fp (~24% lower with *p* = 0.13) or NADH level (Figure 8D). Together, these data indicate that SP-A2 may have a sex-specific role in AM’s NAD(H) redox homeostasis in response to both FA and ozone exposure for males and to ozone exposure for females.

### 3.3. Optical Redox Imaging Detects AM Heterogeneity within an Individual Mouse

**Figure 9:**
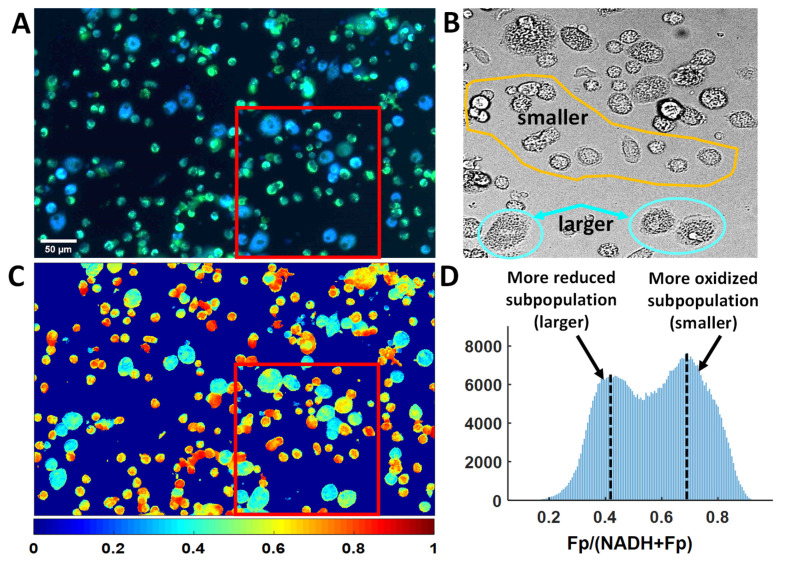
ORI detects AM redox heterogeneity within a male KO mouse with prior ozone exposure. (**A**) A representative composite image of NADH (blue) and Fp (green); (**B**) The white light image of an enlarged area outlined by the red frames in (A) and (C), with some larger and smaller cells encircled in cyan and yellow, respectively; (**C**) The redox ratio image corresponding to the image in (A), displaying two distinct redox subpopulations readily discerned by their pseudo-colors, i.e., cyan (more reduced state, redox ratio ~0.4) vs yellow-orange (more oxidized state, redox ratio ~0.7). The color bar indicates the redox ratio ranging from 0 to 1; (**D**) The histogram of the redox ratios from the image in (C), clearly shows a bimodal redox state, representing the mixture of a more reduced subpopulation and more oxidized subpopulation, respectively. The x-axis represents the redox ratio (Fp/(NADH+Fp)); y-axis represents the number of pixels having a specific redox ratio; dotted line represents the redox ratio of each subpopulation.

**Figure 10:**
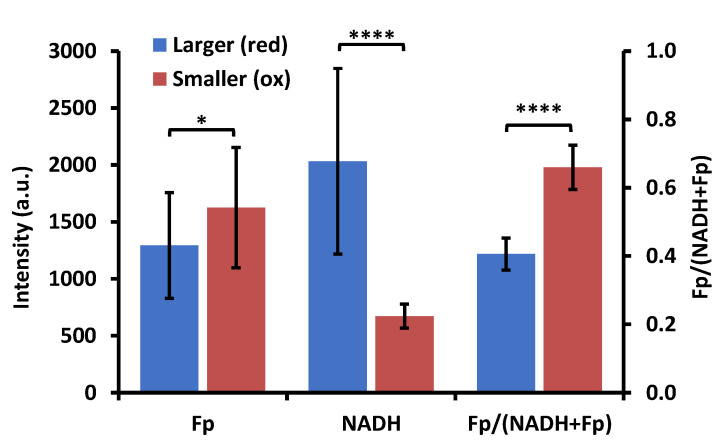
Quantified results for all three redox indices based on 30 cells per subpopulation selected from the four fields of view included those in Figure 9 and Figure S1. * *p* < 0.05, **** *p* < 0.0001.

The AM sample from one ozone-exposed male KO mouse (mouse ID 12273) was considered an outlier and its ORI data were omitted from the previous redox group analysis. However, an initial observation of the AM sample from this mouse revealed something very interesting: There existed cells with two distinct sizes (larger and smaller cells), vastly different from all other AM samples. We acquired images of 18 FOVs with each containing more than 100 cells. From both the composite image of NADH and Fp (Figure 9A) and the white light image (Figure 9B, an enlarged section outlined by the red frame of Figure 9A), one can clearly see the size differences in a typical FOV. Figure 9C is the redox ratio image of the same FOV (redox ratio images from 3 additional FOVs and their corresponding histograms are shown in Figure S1). These redox ratio images as well as their corresponding histograms clearly depict a bimodal distribution, indicating two distinct redox subpopulations of AM despite of some overlap. We quantified the redox indices of these two subpopulations by selecting 30 AM of the larger size (~20–30 μm) and 30 AM of the smaller size (~10 μm) from these four FOVs (Figure 9 and Figure S1). Each cell was one region of interest (ROI) (in cases where it was difficult to draw ROI for one single smaller cell, a cluster of smaller cells were included in the ROI). The mean values of the redox indices of the larger and smaller AM averaged across all selected cells are shown in Figure 10. Student’s *t*-test analysis showed that the redox differences between the two subpopulations are highly significant (*p* < 0.0001 for NADH and the redox ratio and *p* = 0.013 for Fp). These data indicate that AM redox heterogeneity can occur within the same mouse, as shown here, where the larger cells were in a more reduced state (lower Fp, higher NADH, and lower redox ratio), and the smaller cells were in a more oxidized state.

## 4. Discussion

Ozone has been shown to be a pulmonary irritant for several species including human, monkey, and rodents, and it can cause oxidative stress in alveolar macrophages [37,45,49,50,51,52]. Ozone exposure results in oxidative stress via direct action on its target macromolecules or free radicals formed by peroxidation of lipids and induces cellular and molecular alterations and human diseases including pulmonary injury. Ozone inhalation-induced oxidative stress activates both cytotoxic/pro-inflammatory (M1) and wound repairing/anti-inflammatory (M2) macrophages in the lung [49,53]. Macrophage activation is characterized by metabolic shifts and the mitochondria are central hubs in inflammatory macrophage activation [6]. Ozone can accelerate both peroxidative and glycolytic metabolisms in AM [54,55]. 

The NAD(H) redox potential NAD^+^/NADH regulates many enzymatic reactions in cell metabolism including glycolysis, the TCA cycle, respiration, and lipid metabolism. The NAD(H) redox status is intricately related to the balance of cellular reductants and oxidants [19,20,56]. The entanglement of the NAD(H) redox status, sex, and innate immune molecules, such as SP-A2 in AM, has not been reported before, and this was the focus of the present study. We employed optical redox imaging, which is also sensitive to metabolic changes, to study the NAD(H) redox response of a well-established bronchoalveolar macrophage model from mice exposed to ozone (more than 80% of the cells in BAL are macrophages [1,2]). Our results indicate that an ozone exposure of 2 ppm for 3 h resulted in a significantly lower NADH level and higher optical redox ratio in AM from male SP-A2 mice. An early study measuring the metabolite concentrations coupled with the near-equilibrium reactions of β-hydoxybutyrate dehydrogenase showed that the mitochondrial NAD^+^/NADH ratio in AM decreases significantly after exposure to 1 ppm O_3_ for 5 h in vitro [57]. However, the quantification of the redox ratio in that study relied on the assumption that mitochondrial pH remains constant before and after ozone exposure, which might not be valid. Ozone has been shown to oxidize NADH directly in vitro [58] and ROS have been shown to increase the optical redox ratio [19] or the NAD^+^/NADH ratio [59]. While more studies are needed to further confirm the effects of ozone on the cellular NAD(H) redox status, the more oxidized optical redox ratio reported here is consistent with the known effects of the ability of ozone to generate ROS and exhibit oxidation effects. In the present study, we also observed a higher mitochondrial ROS level corresponding to a higher optical redox ratio in AM from SP-A2 male mice than that from female mice after ozone exposure. 

In addition, we report here the effects of ozone exposure on the NAD(H) redox status in the absence of the innate immune molecule, SP-A2, and on the Fp signal, neither of which has been reported before. The Fp signal mainly originates from the oxidized FAD moiety in pyruvate dehydrogenase (PDH) and α-ketoglutarate dehydrogenase (α-KGDH) in the TCA cycle and the electron-transfer flavoprotein in the electron transport chain, accounting for the majority of the Fp signal while the rest is not substrate reducible and is insensitive to redox metabolism [21,25,60,61,62]. The Fp signal is commonly observed to change in opposite direction of NADH, which may be explained by the conjugation of the two redox pairs NAD^+^/NADH and FAD/FADH_2_ in PDH and α-KGDH in the TCA cycle. Higher NADH would result in more FADH_2_ and less FAD and vice versa. Although further studies are needed to identify the exact factors contributing to Fp signal variation as observed in this study, a significantly higher Fp signal upon O_3_ exposure in AM from KO mice may indicate a lower NADH level in mitochondria, presumably correlating with a higher level of ROS and oxidative stress. Note that although the NADH signal detected by ORI can be contributed by cytosolic NADH and NADPH as well, these concentrations are expected to be much lower than NADH in mitochondria and ORI mainly reflects mitochondrial NADH [22,63]. Nonetheless, simultaneous increase or decrease of NADH and Fp signals can indicate a change in mitochondrial densities within cells, induced by genetic and/or environmental factors. In contrast, the optical redox ratio is insensitive to mitochondrial density change and reflects the redox balance. A higher optical redox ratio upon ozone exposure in AM from male SP-A2 mice and from KO mice also indicates an increased mitochondrial bioenergetic activity/oxidative phosphorylation [30,64]. 

The metabolism differs between M1 (usually pro-inflammatory) and M2 (usually anti-inflammatory) macrophages, and macrophages can switch from an oxidative phosphorylation metabolic profile (M2) to a glycolysis profile (M1), and vice versa [7,9]. Ozone inhalation-induced oxidative stress can induce both M1 and M2 phenotypes in AM [49,53], and M2 macrophages can contribute to chronic inflammatory lung diseases as well [65]. A lower optical redox ratio (more reduced state) has been associated with enhanced glycolysis and M1 macrophages while a higher optical redox ratio (more oxidized state) has been associated with M2 macrophages [30]. A similar relationship between the optical redox ratio and glycolysis/OXPHOS balance was also found in T cell activation [66]. Therefore, the observed increased redox ratio of AM after ozone exposure indicates that O_3_ might have induced an M2 phenotype in AM from SP-A2 males, whereas, in AM from SP-A2 females this was not or less the case since no statistically significant changes in the redox ratio were observed (Figure 5). However, in the absence of SP-A2, AM from both male and female mice became significantly oxidized and likely assuming an M2 type activation upon ozone exposure, without sex differences (Figure 6). Admittedly, measurements of M2 macrophage biomarkers (e.g., arginase 1 and chi313 [65,67]) are needed to confirm or disapprove this interpretation.

ORI provides a quick imaging tool to probe macrophage activation of single cells. The interesting bi-modal redox status with both higher and lower redox ratio of the two cell subpopulations observed in AM from a male KO mouse after ozone exposure (Figure 9, Figure 10 and Figure S1) indicates that a heterogeneous macrophage activation can occur in the same BAL sample. While the subpopulation with a higher redox ratio (the smaller cells) is consistent with an anti-inflammatory M2 type activation observed in AM from the other mice, the subpopulation with a lower optical redox ratio (the larger cells) indicates a possible pro-inflammatory M1 type activation (Figure 9, Figure 10 and Figure S1). Based on gene expression analysis, both pro-inflammatory and anti-inflammatory macrophage activation was found in BAL macrophages from human patients of idiopathic pulmonary fibrosis and in an in vitro murine alveolar macrophage model exposed to iron accumulation [1]. Although the cause of this bi-modal redox status in AM in the current study is unknown, the importance of this observation is that this can indeed occur in a single animal, and therefore imaging the NAD(H) redox status in AM could serve as an effective tool to more accurately determine the relative activation of macrophages in terms of the pro- and anti- inflammatory status. Once different subpopulations can be established by ORI on the single cell level, it would be interesting to perform gene expression analysis of subpopulations after cell sorting based on optical redox ratio or NADH signals [68].

In this study, we observed no significant NAD(H) redox differences between AM from male and female mice, either with or without SP-A2, exposed to filtered air (Figure 5 and Figure 6). In contrast, sex differences became evident after ozone exposure in SP-A2 mice, where a more oxidized redox state (a higher redox ratio) and a higher ROS signal were observed in AM from males compared to females (Figure 5 and Figure 7). However, in the absence of SP-A2, no sex differences were observed in the NAD(H) redox status in response to ozone (Figure 6), indicating a role of SP-A2 in the observed sex-dependent differences. Sex dependence of redox features in cells and tissues has been reported in the literature [69,70]. Females have been found to have more antioxidant capacity and greater resistance to oxidative stress [69,71]. Although this is the first time that SP-A and sex dependent differences have been observed in the NAD(H) redox status of AM, sex differences in AM in response to SP-A and/or O_3_ have been seen in other readouts. Consistent with the present findings, previous studies observed that the miRNome of SP-A2 female mice did not change significantly in response to ozone-exposure whereas the AM miRNome in SP-A2 males did [43]. A comparable finding of sex-dependent cellular response after ozone exposure was observed in lung alveolar epithelial type II cells. The miRNome in these cells from SP-A1 female mice did not change significantly but it did in males [72]. Together, these support the notion that at the cellular level females are better prepared to handle oxidative stress in the presence of the innate immune molecule, SP-A2. 

The lower mitochondrial NADH in male KO compared to SP-A2 mice (Figure 8A) suggests a higher bioenergetics activity and likely a higher ROS, indicating that oxidative stress may be expected when SP-A2 is not present. Thus, the present results are consistent with a possible protective role of SP-A2 by modulating the NAD(H) redox status and the oxidative stress after ozone exposure. In fact, it has been previously postulated that SP-A has an antioxidant role in the lung by scavenging ROS [73] or possibly regulating NADPH oxidase activity in human monocyte derived macrophages [74] which may or may not capture the in vivo effect of the ozone-induced oxidative stress in the lungs. It has been shown that the tissue-specific alveolar macrophages differ from monocyte-derived macrophages in response to injury [75,76]. In contrast, SP-A is shown to induce reactive oxygen species and cytokine responses by local AM in response to infection [77]. Moreover, in an earlier report where the bronchoalveolar lavage proteome was studied in response to ozone-exposure in the presence or absence of SP-A, the KO under control conditions (i.e., filtered air exposure) were found to exhibit a response pattern similar to that observed in the wild type mice after ozone exposure [78]. The similarity in response between SP-A KO mice exposed to filtered air and wild type mice exposed to ozone indicates that in the absence of SP-A, KO mice may be in a chronic oxidative stress state. Thus, the collective data provide support for a role of SP-A as antioxidant and the present findings, in particular, indicate that this may occur via NAD(H) redox changes in a sex-specific manner.

AM sex-dependent differences have also been observed using insults other than ozone, such as infection. These include, following *K. pneumoniae* infection, SP-A variant and sex-dependent differences in AM gene expression [79], as well as in lung function [42] and survival [41]. Females have been shown to have an overall better outcome in response to a single insult (i.e., infection). This may be due in part to a better-maintained redox homeostasis in females as shown in the present study. Of interest, although ozone, by itself under the experimental conditions used here, is not lethal, if mice after exposure to ozone are also infected with *K. pneumoniae*, females show a lower survival compared to males [33,34]. The underlying mechanisms, except that sex hormones play a role in this differential survival [80], are currently unknown.

The current study has some limitations. First, we have used female mice with synchronized estrous cycles to perform the study. However, the phase of the estrous cycle was not determined. Because the sex hormone profile varies throughout the estrous cycle and can affect lung function and inflammation [81,82] and possibly SP-A2 activity following ozone exposure, future determination of the estrous cycle phase and the sex hormone profile will be valuable. Second, the study of mice exposed to ozone may not entirely reflect the situation in human subjects. Third, we performed this study without virus or bacterial infection. To ultimately understand the molecular links between the role of the NAD(H) redox status, sex, and SP-A2 in innate immunity and relevant pathologies, we may utilize virus/bacteria infected epithelial cells and mouse models in the future. We may also expand the study to human BAL samples.

As a final note, the present findings are potentially relevant to the current COVID-19 pandemic and may provide insight into the role of innate immune molecules, such as SP-A, in oxidative stress and viral host defense and how this may differ between men and women. Correlation between oxidative stress markers and severity of many viral diseases, such as hepatitis C and influenza is well established, but clinical data for SARS-CoV (severe acute respiratory syndrome coronavirus) are limited. However, animal model studies of SARS-CoV infection have shown that activation of ROS production along with innate immunity, transcription factors such as NF-κB, as well as a disturbance in anti-oxidative defense results in an exacerbated pro-inflammatory host response and severe lung injury [83,84]. SP-A has been also shown to activate NF-κB [85], and under baseline conditions and in response to infection with and without prior ozone exposure [32,39,43,86] to affect some of the signaling pathways observed in SARS-CoV infection. Thus, the published information indicates a role of SP-A in various signaling pathways including anti-oxidant pathways that may be relevant to SARS-CoV-2 (severe acute respiratory syndrome coronavirus 2) as well, and the present data indicate that the SP-A-mediated NAD(H) redox status may be one of the mechanisms.

The SARS-CoV-2 causes acute lung injury that may develop into life-threatening acute respiratory distress syndrome in elderly individuals. However, the majority of young patients recover, indicating that protective host responses may be operational to combat the viral infection. Though males and females get infected with SARS-CoV-2 at the same rate, males are at higher risk for severe disease and death, independent of age [87]. The underlying mechanisms and whether it is due to gender and/or sex differences is not known at this time. Given the sex-specific and the SP-A genotype specific outcomes in animal models [41,42,79,86,88] and the important role of innate immunity in providing the first line of defense, it is highly likely that the genetics of the innate immunity molecule, SP-A, contribute to and/or may partly explain the differential outcome in COVID-19 patients under different lung microenvironments [89,90]. The genotype- and sex-dependent effect of SP-A on NAD(H) redox status and oxidative stress/inflammatory pathways may be of clinical relevance in COVID-19 patients and SP-A may be considered as a potential therapy. The rationale for this potential therapy is based on the information where SP-A is shown to modulate pathways either under baseline conditions [32,40] or in response to infection and/or ozone exposure [43,86,88] that are deemed important in COVID-19 disease [91]. These include, among others, the Nrf2 and the acute phase response that includes NF-κB and the IL-6.

In addition, since the pathology of COVID-19 patients is characterized by excessive inflammatory macrophage activation [92], we would expect ORI to be a useful tool. It can be used to evaluate the severity and heterogeneity of macrophage activation in BAL of alveolar macrophages from COVID-19 patients. This may help to better understand the SARS-CoV-2 induced inflammatory macrophage activation and develop interventional approaches to mitigate the inflammation in COVID-19 patients.

## 5. Conclusions

Sex is a significant factor for human health, including immune responses to environmental insults such as air pollution, infection, and others. Our study indicates that the NAD(H) redox status of alveolar macrophages shifts to a more oxidized state after exposing mice to ozone and SP-A2 countermeasures the ozone oxidation effect in females but not in males. This, to the best of our knowledge, is the first time to show ozone-induced NAD(H) redox change of mouse AM occurring in a sex-dependent and SP-A2 gene-dependent manner. The findings from this study indicate that SP-A genetics and the NAD(H) redox status may be entangled in innate immunity and inflammation in a sex-dependent manner. These findings point to possibly new directions for understanding and treating COVID-19.

## Supplementary Materials

The following are available online at https://www.mdpi.com/2076-3921/9/10/915/s1, Figure S1: ORI-detected AM redox heterogeneity within a male KO mouse with prior ozone exposure shows a bimodal redox state. Table S1: The mean redox indices of the studied mice exposed to filtered air (FA, as control group) or Ozone (O_3_).
