# Supplementary material for: Sex and SP-A2 Dependent NAD(H) Redox Alterations in Mouse Alveolar Macrophages in Response to Ozone Exposure: Potential Implications for COVID-19
Source: Antioxidants (Basel). 2020 Sep 25;9(10):915. doi: 10.3390/antiox9100915 (PMC7601279; doi:10.3390/antiox9100915)
Supplement: Supplementary file 1 [file antioxidants-09-00915-s001.pdf]

Supplementary Materials

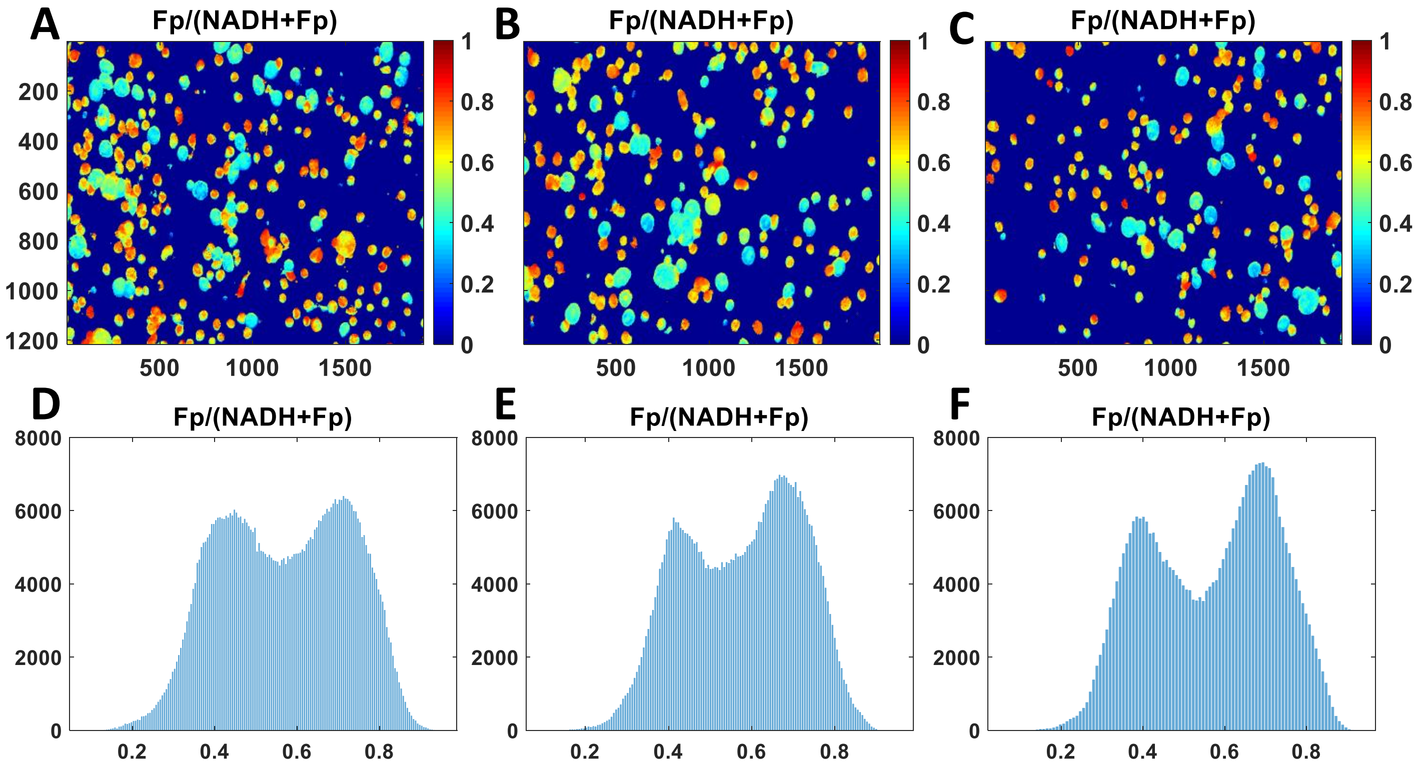

Figure 1S. ORI-detected AM redox heterogeneity within a male KO mouse with prior ozone exposure shows a bimodal redox state. (A)-(C) Typical redox ratio images displaying two distinct redox subpopulations readily discerned by their pseudo-colors and their corresponding histograms (D)-(F).

Table S1. The mean redox indices of AM from the studied mice exposed to filtered air (FA, as control group) or Ozone (O_3)

SP-A2 mice	Mouse ID	Exposure	Gender	Fp	NADH	Fp/(NADH+Fp)
SP-A2 (M, FA)	10661	FA	M	1239	754	0.574
SP-A2 (M, FA)	10662	FA	M	1148	700	0.565
SP-A2 (M, FA)	10665	FA	M	1142	699	0.564
SP-A2 (F, FA)	10634	FA	F	922	616	0.552
SP-A2 (F, FA)	10635	FA	F	989	730	0.526
SP-A2 (F, FA)	10655	FA	F	1000	710	0.529
SP-A2 (M, O_3)	10739	O_3	M	1226	569	0.652
SP-A2 (M, O_3)	10743	O_3	M	1376	589	0.652
SP-A2 (M, O_3)	10744	O_3	M	1433	608	0.650
SP-A2 (M, O_3)	10754	O_3	M	1368	609	0.646
SP-A2 (M, O_3)	10755	O_3	M	1116	581	0.596
SP-A2 (F, O_3)	10736	O_3	F	1026	605	0.574
SP-A2 (F, O_3)	10737	O_3	F	1101	657	0.564
SP-A2 (F, O_3)	10741	O_3	F	1099	590	0.591
SP-A2 (F, O_3)	10742	O_3	F	1374	680	0.612

KO mice	Mouse ID	Exposure	Gender	Fp	NADH	Fp/(NADH+Fp)
KO (M, FA)	10731	FA	M	905	592	0.567
KO (M, FA)	10733	FA	M	864	498	0.584
KO (M, FA)	12281	FA	M	1031	562	0.593
KO (M, FA)	13638	FA	M	944	619	0.558
KO (F, FA)	12277	FA	F	928	672	0.524
KO (F, FA)	12279	FA	F	1103	604	0.594
KO (F, FA)	12287	FA	F	898	538	0.577
KO (M, O_3)	10722	O_3	M	2358	757	0.717
KO (M, O_3)	10723	O_3	M	1928	712	0.687
KO (M, O_3)	12272	O_3	M	1470	601	0.666
KO (M, O_3)	13633	O_3	M	1715	684	0.669
KO (F, O_3)	12283	O_3	F	1587	748	0.614
KO (F, O_3)	12284	O_3	F	1189	571	0.621
KO (F, O_3)	12285	O_3	F	1706	628	0.685
KO (F, O_3)	12286	O_3	F	1577	616	0.664
*KO (M, O_3)	12273	O_3	M	1358	956	0.564
* An outlier						
